# Protective Effect of Resveratrol on Benzo(a)Pyrene Induced Dysfunctions of Steroidogenesis and Steroidogenic Acute Regulatory Gene Expression in Leydig Cells

**DOI:** 10.3389/fendo.2019.00272

**Published:** 2019-04-30

**Authors:** Bhaswati Banerjee, Supriya Chakraborty, Pratip Chakraborty, Debidas Ghosh, Kuladip Jana

**Affiliations:** ^1^Division of Molecular Medicine, Bose Institute, Calcutta Improvement Trust Scheme VIIM, Kolkata, India; ^2^Department of Infertility, Institute of Reproductive Medicine, Kolkata, India; ^3^Department of Bio-Medical Laboratory Science and Management, Vidyasagar University Midnapore, Midnapore, India

**Keywords:** B(a)P, leydig cell, ROS, StAR, p38 MAPK, SF1

## Abstract

Benzo(a)pyrene [B(a)P] is the toxic environmental Polycyclic Aromatic Hydrocarbon (PAH), that exerts male reproductive dysfunctions. In this study the molecular mechanism of B(a)P induced Leydig cell steroidogenic dysfunctions and its protective mechanism of action with a natural Aryl hydrocarbon receptor (AhR) antagonist and anti-oxidant, Resveratrol (Res) has been investigated. B(a)P exposure induced ROS mediated steroidogenic imbalance via activation of p38MAPK and repression of testosterone level as well as other steroidogenic enzymes like CYPIIA1, 3β-HSD, 17β-HSD expressions. B(a)P exposure decreased StAR protein expression along with increased DAX-1, a transcriptional repressor of *StAR* gene. Along with that B(a)P decreased the expression of SF-1 that acts as a transcriptional inducer of StAR gene expression. The study has established Resveratrol as a potential agent combating the deleterious effect of B(a)P on Leydig cell steroidogenesis. Resveratrol treatment resulted significant protection against B(a)P by scavenging ROS and modulating the transcriptional regulation of anti-oxidant enzymes. Furthermore, Resveratrol also prevented stress kinase like p38 MAPK activation and increased StAR protein expression through the reduction of DAX-1 expression. Moreover, the testosterone production was efficiently restored with Resveratrol treatment. ChIP assay also revealed that resveratrol improved SF-1expression which further increased the *StAR* gene expression. Resveratrol acted efficiently against B(a)P, through its anti-oxidative properties as well as inhibits p38MAPK and increased steroidogenesis and StAR expression through the modulation of *SF-1* gene expression.

## Introduction

Leydig cells comprise the major endocrine compartment of testis and produce testosterone, the major male reproductive hormone, by the process of steroidogenesis. Several investigations have proposed that environmental toxicants induce adverse effects on male reproductive health (1, 2). These environmental toxicants possess endocrine disrupting properties. Thus, they interfere with the process of steroidogenesis and prevent testosterone production (3). B(a)P is an environmental PAH that act as an endocrine disruptor (4). B(a)P is generated in the ambient air from the incomplete combustion of organic fuel. B(a)P induce several health hazards and counted as Group I carcinogen. Our previous findings revealed that B(a)P exposure resulted deleterious effects on male reproductive system and imparted steroidogenic dysfunctions (5, 6) at low to moderate exposure. B(a)P induced biological effects are mediated through the Aryl hydrocarbon Receptor (AhR). Ligand bound AhR acts as a ligand-activated transcription factor and induces the transcription of cytochrome enzymes associated with phase I xenobiotic and drug metabolism (like CYP1A1). But the mode of action of B(a)P in Leydig cells is not properly elucidated.

The major function of testicular Leydig cells is to produce the male hormone testosterone, under the influence of Luteinizing hormone (LH). The first and rate-limiting step in steroidogenesis is the transfer of cholesterol across the inner membrane space from the outer mitochondrial membrane to the inner mitochondrial membrane, a process dependent on the action of Steroidogenic Acute Regulatory (StAR) protein (7). The next step involves the oxidative cleavage of the side chain of the substrate cholesterol by CYP11A1, a mitochondrial cytochrome P450 oxidase and its conversion to pregnenolone. In the next step, pregnenolone is further oxidized by the CYP17 group of enzymes in the endoplasmic reticulum to yield a variety of C-19 steroids. In addition; the 3-hydroxyl group is oxidized by 3β HSD to produce androstenedione. In the final step, the androstenedione is reduced by 17β HSD to yield testosterone (7, 8).

In the initial stage of steroidogenesis, cholesterol needs to be transferred from the outer membrane to the inner membrane of mitochondria since the CYP11A1 enzyme is located on the inner mitochondrial membrane. The aqueous space between the outer and inner mitochondrial membrane acts as a barrier to cholesterol transfer because of its hydrophobic nature. As a result, diffusion of cholesterol through this water barrier is very slow and is not able to supply sufficient substrate for adequate testosterone production. Therefore, the mitochondrial cholesterol transfer via StAR becomes the vital step of testosterone biosynthesis (9). Reports suggest that StAR can be regulated by endogenous and exogenous agents, including environmental toxins (10). It is well-demonstrated that environmental PAHs greatly affect LH stimulated Leydig cell testosterone production (3, 4, 11). Testosterone biosynthesis is known as steroidogenesis and that in turn greatly affects spermatogenesis. The mechanism of B(a)P induced down regulation of StAR expression is not yet explained. The aim of our study was to investigate the mode of action of B(a)P in the inhibition of StAR protein expression and also the subsequent antagonistic effect of Resveratrol (Res) on it.

StAR expression and steroidogenesis involves transcriptional induction. The promoter for *StAR* has been sequenced for different mammalian species (rat, mouse, human) (12–14) and they share extensive homology. However, *StAR* gene promoter lacks a consensus cAMP response element (CRE) and, as such, resembles the promoter of several steroid hydroxylase genes that are regulated by cAMP (15). Transcriptional regulation of the StAR gene, in the absence of a consensus CRE, has been demonstrated to be mediated by multiple DNA elements that provide recognition motifs for sequence-specific transcription factors. These include steroidogenic factor 1 (SF-1), CCAAT/ enhancer binding protein (C/EBP), GATA, sterol regulatory element binding protein (SREBP), SP1, CREB/CRE modulator protein (CREM), the activator protein 1 (AP-1) family (Fos and Jun), DAX-1 (dosage-sensitive sex reversal-adrenal hypoplasia congenital critical region on the X-chromosome) etc. (16, 17). Therefore, it is well-defined that the proximal region of the StAR promoter is the preferential binding site for several transcription factors and they play crucial roles in controlling *StAR* gene expression.

It has been confirmed that cAMP modulates StAR through a PKA (protein kinaseA) mediated event. PKA activation can regulate StAR directly and indirectly. Indirectly, PKA can induce transcriptional activation by phosphorylating steroidogenic factor 1 (SF1), a transcription factor commonly known to regulate StAR expression. There are multiple SF-1 sites throughout the mammalian StAR promoter (18). These cellular events trigger activation of two transcription factors i.e., SF1 and DAX 1 those are known to be involved in the regulation of *StAR* gene expression. SF1 up-regulates the expression of StAR protein and several other steroidogenic enzymes (19), while DAX1, that blocks SF-1 activity thus down regulates StAR expression (20). From the previous reports it can be concluded that the ratio between these two transcription factors determines whether the combined effect will either enhance or inhibit steroidogenesis in different steroidogenic cell types and at different stages of development (20).

Recent findings indicate that p38 MAPK regulates steroidogenesis through transcriptional repression of *StAR* gene (21). Our previous findings established that B(a)P induced testicular steroidogenic dysfunction is associated with the activation of p38MAPK and production of oxidative stress (6). Henceforth, we have tried to delineate the molecular interplay behind B(a)P induced repression of StAR expression in Leydig cells.

Several phytochemicals are receiving overgrowing importance as therapeutic targets against several health-related disorders. Our previous findings suggest that resveratrol (Res), a plant polyphenolic compound possesses protective properties against B(a)P induced reproductive dysfunctions (5, 6). To our knowledge, there is no detailed report of the steroidogenic-protective role of resveratrol against B(a)P induced damage in Leydig cells, available in the literature so far. Therefore, this is the first study demonstrating the protective potential of resveratrol and its mechanism of action in normal Leydig cells after acute B(a)P exposure.

Herein we have investigated the putative mechanism of action of B(a)P induced Lyedig cell steroidogenic dysfunctions and the protective role of resveratrol. Our study has validated the *in-vivo* findings in *in-vitro* system to investigate the molecular interplay induced by B(a)P in Leydig cell steroidogenesis and its possible protection with resveratrol.

## Materials and Methods

### Materials

B(a)P, 3,5,4′-trihydroxy-trans-stilbene (resveratrol), 2, 7-dichlorofluorescein diacetate (DCF-DA), DAPI, NAC (N Acetyl Cysteine) all secondary antibodies (HRP and/or FITC tagged) were purchased from Sigma Chemical Company, St. Louis, USA. Testosterone ELISA kit was purchased from Calbiotech (Spring Valley, CA, USA). MTT cell proliferation assay kit was procured from HIMedia, India. TRIZOL reagent was purchased from Invitrogen (Carlsbad, CA, USA.). Verso cDNA synthesis kit was purchased from Thermo Fisher (Waltham, MA, USA). Powerup SYBR green Master Mix (Applied Biosystems, CA, USA). Antibodies for StAR, 3βHSD, 17βHSD, CYP11A1, SF1, DAX1, AhR and β actin were purchased from Santa Cruz Biotechnology (Santa Cruz, CA, USA). Anti- CYP1A1, phospho p38 MAPK, and p38 MAPK antibodies were procured from Abcam (Cambridge, MA, USA). All the chemicals and reagents were analytical grade.

### Methods

#### Animals and Drug Treatment

Adult male Wistar rats (*Rattus norvegicus)*, 8 weeks of age; 150–200 g body weight were housed in a climate-controlled (22 ± 2°C) animal room at a constant 12/12-hr light/dark cycle, with free access to food and water. All procedures were performed in accordance with the protocols approved by the Institutional Animal Ethical Committee (IAEC/BI/30/2015). Four animal groups were maintained. For each group n = 10 were maintained. Group 1: control animals were gavaged the same volume (0.2 ml) of vehicle (Corn oil) for 60 days, Group 2: animals received resveratrol (Res) at the dose of 50 mg/kg of body weight daily through oral gavage for 60 days, Group 3: animals received B(a)P at the dose of 5 mg/kg of body weight daily through oral gavage for 60 days and Group 4: animals were gavaged B(a)P (5 mg/kg) along with resveratrol (50 mg/kg of body weight) for 60 days. We have selected the single dose for both B(a)P and resveratrol from our previous studies, as these have been the most effective doses under our experimental set up. Animals receiving vehicle or 50 mg/kg body weight resveratrol alone did not show any adverse side effects. Euthanasia was performed by decapitation under sodium pentobarbital anesthesia, and the efforts were made to minimize the pain and sufferings to the animals. Testes were collected after sacrifice of the animals.

#### Ethics Statement

The study was performed under strict accordance with the protocols of the National Institute of Health guidelines for the Care and Use of Laboratory Animals (NIH publication No. 85–23 revised 1985: US Department of Health, Education and Welfare, Bethesda, Maryland, USA). The experimental outline also met the National Guidelines on the Proper Care and Use of Animals in Laboratory Research (Indian Science Academy, New Delhi, India) and the protocol was approved by the Institutional Animal Ethics Committee (IAEC) of Bose Institute, Kolkata, India (Approval No. IAEC/BI/30/2015).The animal breeding and experimental facility are registered with the Committee for the Purpose of Control and Supervision of Experiments on Animals (CPCSEA), Ministry of Environment and Forest and Climate Change, Government of India.

#### Leydig Cell Isolation

Decapsulated testes of rats from different study groups were incubated with dissociation buffer (DMEM/F12 containing 0.1% BSA and 0.25 mg/ml collagenase) at 34°C in a shaking water bath for 15 min. After dissociation the seminiferous tubules were removed by filtration through 100 μm nylon mesh. The filtrate was centrifuged (250 × g for 10 min), the pellet was resuspended in buffered Hanks' balanced salt solution, and the suspension was mixed with iso-osmotic Percoll in Hanks' balanced salt solution. After centrifugation (20,000 × g for 60 min at 4°C), fractions were collected and washed with dissociation buffer (11). Leydig cell purity was assessed by cytochemical staining for 3β HSD. The cell purity consistently was about 85%.

#### TM3 Cell Culture

TM3 normal mouse Leydig cell line was obtained from the National Center for Cell Science (NCCS, India). Cells were maintained and propagated in DMEM with various supplements as suggested by NCCS. The cells were incubated in a humidified incubator at 37°C with 5% CO_2_ and were exposed to B(a)P, resveratrol or other reagents when confluency reached 50% (11). Doses for *in-vitro* study of the effect of B(a)P and resveratrol were initially based on previous reports (11, 22).

#### Assay of Testosterone Concentration

Testosterone concentrations were determined in cultured cell media using Calbiotech ELISA kit according to the manufacturer's instructions. The sensitivity of the assay is 0.075 ng/ml. The intra assay co-efficient of variation is 2.9% and inter assay coefficient of variation is 3.4%.

#### MTT Cell Viability Assay

For viability assays, cells were seeded on 96-well-plates at a density of 0.5 × 10^5^ cells/well. Cell viability was measured by using the MTT cell proliferation assay kit (HIMedia). Absorbance was read at 570 nm on a microplate reader (MolecularDevices). Cell viability was expressed as a percentage of the control culture.

#### Immuno-Cytochemistry

For fluorescence imaging, the cells were fixed with 3.7% formaldehyde and permeabilized with 0.1% TritonX-100. Then, cells were incubated with the appropriate primary antibody in 1% BSA at 4°C for overnight. For secondary antibody reaction, cells were incubated with an appropriate fluorescence-conjugated secondary antibody at room temperature. Cell nuclei were counterstained with DAPI (4′6-diamidino-2-phenylindole). The samples were mounted on clean glass slides using Vecta Shield mounting media (Vector Laboratories, Burlingame, CA) and visualized under the confocal microscope (SP8 Leica, Germany). Images were quantified using this formula to calculate the corrected total cell fluorescence (CTCF). CTCF = Integrated Density–(Area of selected cell X Mean fluorescence of background readings).

#### Intracellular ROS Production in Leydig Cells

Intracellular ROS production was estimated by using 2, 7-dichlorofluorescein diacetate (DCF-DA) as a probe according to the method of Kuo and Tang (23). Briefly, 100 μl of cell lysate was incubated with the assay media (20 mM tris-HCl, 130 mM KCl, 5 mM MgCl_2_, 20 mm NaH_2_PO_4_, 30 mM glucose and 5 μM DCF-DA) at 37°C for 15 min. The formation of DCF was measured at the excitation wavelength of 488 nm and emission wavelength of 510 nm for 10 min by using spectro-fluorometer (Hitachi) equipped with a FITC filter.

#### Western Blotting Analysis

Cells were lysed in RIPA lysis buffer, supplemented with protease and phosphatase inhibitor cocktail (both from Life Technologies). Proteins from cell lysates (30 μg) were resolved on 10% SDS–PAGE and electro-transferred onto PVDF membrane (Millipore). Membranes were incubated with primary antibodies against StAR, CYPIIA1, 3β HSD, 173β HSD, SF1, DAX1, AhR, CYP1A1, phospho p38MAPK, p38MAPK, and β Actin. Next membranes were incubated with respective secondary antibodies and visualized by ECL detection. β Actin was used as the internal loading control.

#### Reverse Transcriptase PCR and qRT PCR:

mRNA expression of target genes was estimated using semi-quantitative reverse transcriptase PCR method. Briefly, total RNA from cells and/or tissue was extracted with TRIZOL reagent (Invitrogen) and cDNA was synthesized according to the manufacturer's instructions. cDNA was subjected to PCR (30 cycles) in 20μL reaction mixture [10 × PCR buffer, 2.5 mM dNTP, Taq-polymerase 1U, and forward and reverse primers]. The PCR products were resolved by 1% agarose gel electrophoresis and visualized using Ethidium bromide. The primer sequences are stated in the Table 1. Quantitative RT-PCR was performed using specific primers (Table 1) and amplified using Powerup SYBR green Master Mix (Applied Biosystems) in Real time PCR (Applied Biosystems). Results are presented as relative mRNA expression levels calculated using the formula 2^−Δ*CT*^, where ΔCT = ΔCT_target_-ΔCT_reference_ with β *Actin* as the reference gene.

**Table 1:** Sequences of primers for Reverse transcriptase PCR and qRT PCR.

**Primer**	**Forward (5′-3′)**	**Reverse (5′-3′)**
StAR	TTGGGCATACTCAACAACCA	ATGACACCGCTTTGCTCA
3β-HSD	CCGCAAGTATCATGACAGA	CCGCAAGTATCAGACAGA
17β-HSD	TTCTGCAAGGCTTTACCAGG	ACAAACTCATCGGCGGTCTT
CYP11A1	CGCTCAGTGCTGGTCAAAA	TCTGGTAGACGGCGTCGAT
SF1	TCATCCTCTTCAGCCTGGAT	AGGTACTCCTTGGCCTGCAT
DAX1	CACTTGCTCCCAGCTGCTGC	TTGATGAATCTCAGCAGGAA
CYP1A1	CTGGTTCTGGATACCCAGCTG	CCTAGGGTTGGTTACCAGG
AhR	GGGATCGATTTCGAAGACATCAG	AACGCCTGGGAGCCTGGAATCTC
β actin	GGAGATTACTGCCCTGGCTCCTA	GACTCATCGTACTCCTGCTTGCTG
StAR (Mouse)	GGAAGTCCCTCCAAGACTAAAC	AGTCCTAGTGTCTCCTGACTAC
3β-HSD (Mouse)	CACTGGAAGCTGTGTGAAGA	GGGTCAGCACCTGAATAATGA
17β-HSD (Mouse)	TGCAACATTACCTCCGTAGTC	CAGCTCCGATCGTGACATATT
CYP11A1(Mouse)	GTCCTTCAATGAGATCCCTTCC	CCCAATGGGCCTCTGATAATAC
β actin (Mouse)	GACAGGATGCAGAAGGAGATTAC	TCAGTAACAGTCCGCCTAGAA
StAR (qRTPCR)	GGAAGTCCCTCCAAGACTAAAC	ACTCTATCTGGGTCTGCGATA
SF1 (qRTPCR)	CGTCTGTCTCAAGTTCCTCATC	TTTCCTGGGCGTCCTTTAC
SOD1 (qRTPCR)	TCTAAGAAACATGGCGGTCC	CAGTTAGCAGGCCAGCAGAT
SOD2 (qRTPCR)	CTGAGGAGAGCAGCGGTGGT	CTTGGCCAGCGCCTCGTGGT
GPx1 (qRTPCR)	CTCTCCGCGGTGGCACAGT	CCACCACCGGGTCGGACATAC
Catalase (qRTPCR)	GGCAGCTATGTGAGAGCC	CTGACGTCCACCCTGACT
β actin (qRTPCR)	CAGCCTTCCTTCTTGGGTATG	GGCATAGAGGTCTTTACGGATG

#### ChIP Assay

Chromatin immuno-precipitation assay was performed using ChIP assay kit (Millipore) following manufacturer's instructions. Isolated chromatin was precipitated with SF1 antibody. Input DNA, rabbit IgG-pulled DNA served as controls for all the experiments. Immunoprecipitated DNA was then subjected to 40 cycles of PCR using primer pairs specific for StAR promoter region 5′-TGATGCACCTCAGTTACTGG-3′(forward) and 5′-GCTGTGCATCATCACTTGAG-3′(reverse). β *Actin* was used as a nonspecific control for the ChIP experiments. Two percent agarose gel with Ethidium bromide was used to separate and examine PCR products. The results were normalized to the chromatin input of the IP.

#### Statistical Analysis

The results were expressed as mean ± SEM. One-way ANOVA was followed by Dunnett multiple comparison test, except where otherwise indicated. The level of significance was set at ^***^(*P* < 0.001); ^**^(*P* ≤ 0.01–0.001); ^*^(*P* ≤ 0.01–0.05) in respect with the control. Graph Pad Prim 5.0 was used as the statistical analysis software.

## Results

### Resveratrol Prevents B(a)P Induced Testicular Leydig Cell Steroidogenic Dysfunctions via ROS Generation and p38 MAPK Activation

**Figure 1:**
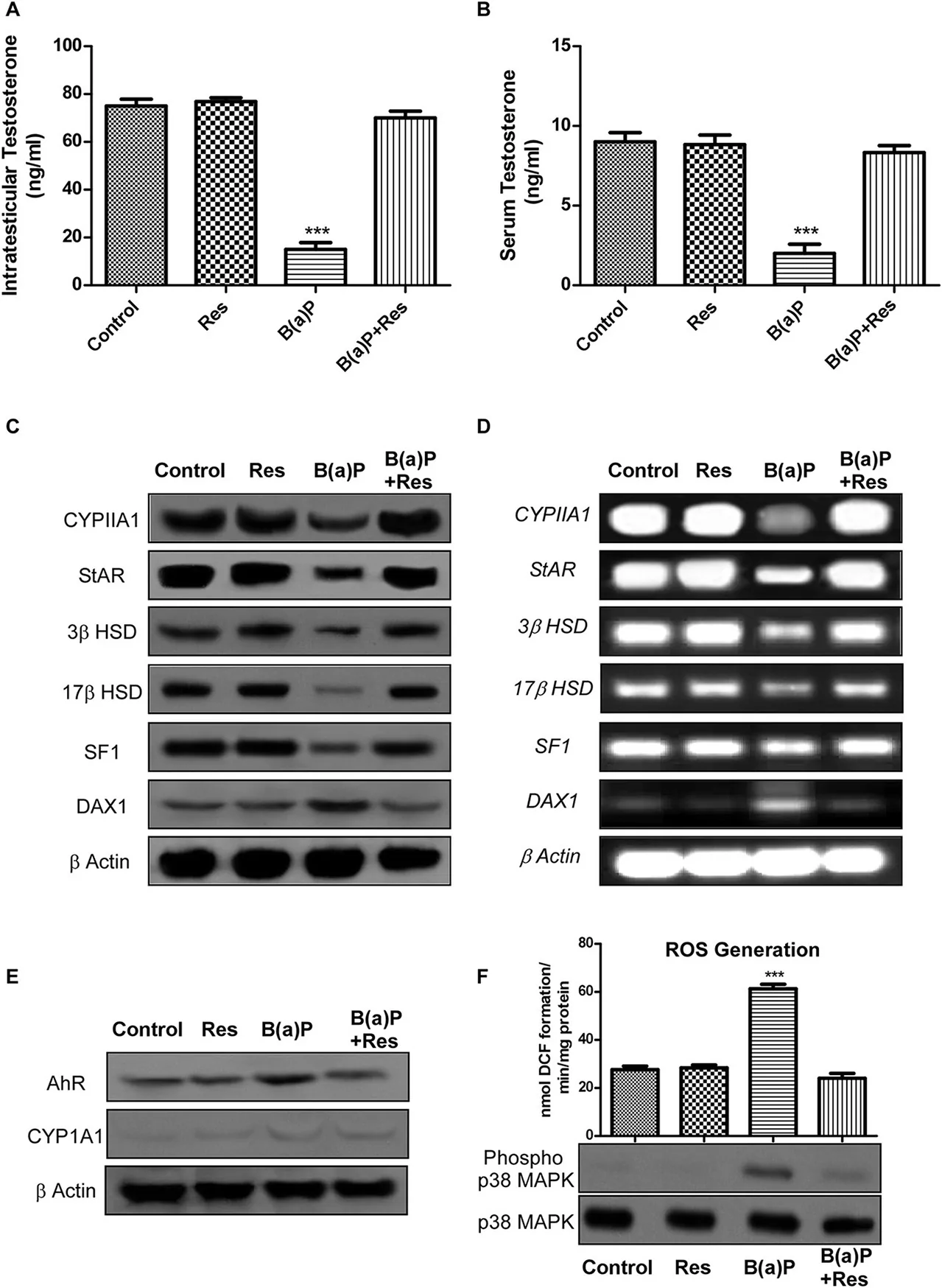
Graphical representation of the level of **(A)** intra-testicular Testosterone (ng/ml) and **(B)** serum Testosterone (ng/ml). **(C)** Western blot and **(D)** RT-PCR of *CYP11A1, StAR, 3*β *HSD, 17*β *HSD, SF1*, and *DAX1* with isolated testicular Leydig cells. **(E)** Western blot of AhR and CYP1A1 with isolated Leydig cells. β Actin was used as the loading control. **(F)** ROS generation in isolated testicular Leydig cells; Western blot of phospho and complete p38 MAPK (lower panel). At least three independent experiments were performed. Histogram is expressing the value of, mean ± SEM. One-way ANOVA was followed by Dunnett multiple comparison test. The level of significance was set at ^***^(*P* < 0.001) as compared with control. B(a)P, Benzo(a)pyrene (5 mg/kg); Res, Resveratrol (50 mg/kg).

Our findings suggested that B(a)P exposure significantly alters steroidogenesis in testis (6). Herein we have investigated the role of resveratrol (Res) as a potent agent restoring Leydig cell steroidogenic functions. Testosterone is the major male reproductive hormone. Circulating testosterone and intra-testicular testosterone levels are the prime indicators for steroidogenic functioning of testis. The findings have shown that B(a)P exposure significantly decreased (*p* ≤ 0.001) testicular and serum testosterone level (Figures 1A,B). Res improved testosterone level in testis and serum (Figures 1A,B). At molecular level CYP11A1, StAR, 3β HSD, and 17β HSD are strongly involved with the process of steroidogenesis. Their translational and transcriptional regulation must be maintained for sustained testosterone synthesis. For the studies primary Leydig cells were isolated from rat testis upon B(a)P and Res treatment. Western blot and RT-PCR with isolated testicular Leydig cells resulted decrease in protein and mRNA expression of major steroidogenic molecules like *CYP11A1, StAR, 3*β *HSD*, and *17*β *HSD* (Figures 1C,D). Res improved the steroidogenic profile of testicular Leydig cells, thus the expression of steroidogenic molecules were improved.

SF1 and DAX1 are two transcription factors, involved with the process of steroidogenesis. SF1 promotes steroidogenesis via StAR promoter whereas DAX1 prevents steroidogenesis via blocking SF-1 activity (20). Findings suggested that B(a)P induced decrease in SF1 and increase in DAX1 expression. Res treatment significantly increased SF1 expression and decreased DAX1 expression (Figures 1C,D) in primary Leydig cells.

B(a)P being a PAH, exerts its biological activity via AhR and metabolized primarily by CYP1A1. The experimental outcomes suggested that Res effectively prevented B(a)P induced increase in AhR expression. Previous findings suggested that primary Leydig cells express very poor level of CYP1A1 (11) as an inbuilt mechanism for protecting cells from toxicant exposure and our findings reconfirmed the fact (Figure 1E).

Oxidative stress results from excessive biosynthesis of Reactive Oxygen Species (ROS), impaired biosynthesis of antioxidants, or a combination of both. ROS is a group of highly reactive oxidizing agents that are involved in several biological processes. But excessive production of ROS damages almost all systems. Balancing ROS and antioxidants is vital for normal testicular functions and sperm fertilization ability (3). Our previous findings suggested that B(a)P induced testicular oxidative stress (6). Res being a potent antioxidant prevented ROS generation in testis. Herein we have investigated the production of ROS in Leydig cells as ROS induces negative impact in Leydig cell steroidogenesis (24). p38MAPK is a significant player behind ROS induced steroidogenic dysfunction (21, 24). Results exhibited that B(a)P induced ROS generation and p38 MAPK activation in isolated primary Leydig cells (Figure 1F). Resveratrol significantly prevented B(a)P induced ROS generation (*p* ≤ 0.001) and also decreased p38 MAPK activation (Figure 1F).

#### B(a)P Prevents Testosterone Production but Does Not Induce Cell Death in TM3 Leydig Cells

**Figure 2:**
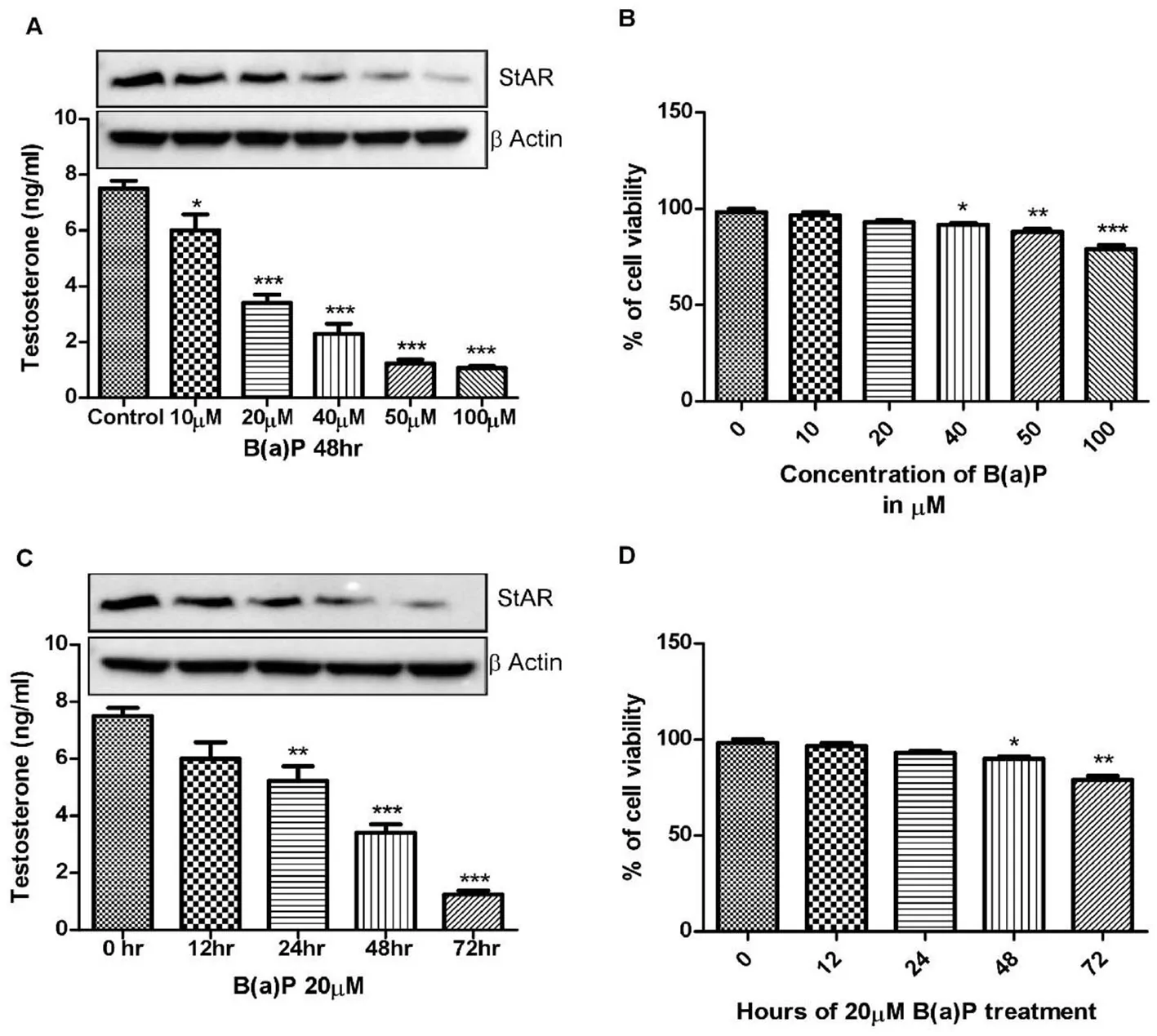
**(A)** Dose dependent effect of B(a)P (10–100 μM) on steroidogenesis in TM3 cells for 48 h. Histogram expressing testosterone level (ng/ml) in media and corresponding western blot of StAR (upper panel). **(B)** Histogram exhibiting cell viability after treatment with increasing concentrations of B(a)P (10–100 μM) in TM3 cells for 48 h. **(C)** Time dependent effect of B(a)P on steroidogenesis in TM3 cells. Histogram expressing testosterone level (ng/ml) in media and corresponding western blot of StAR (upper panel). **(D)** Histogram representing cell viability after treatment with increasing concentrations of B(a)P at different time points with 20 μM B(a)P in TM3 cells. β Actin was used as the loading control. At least three independent experiments were performed. Histograms are expressing the value of, mean ± SEM. One-way ANOVA was followed by Dunnett multiple comparison test. The level of significance was set at ^***^(*P* < 0.001); ^**^(*P* ≤ 0.01–0.001); ^*^(*P* ≤ 0.01–0.05) as compared with control. B(a)P, Benzo(a)pyrene.

After obtaining the primary data in isolated Leydig cells, to study the mechanism of action of B(a)P induced steroidogenic dysfunction, we moved toward *in-vitro* experiments. The principal preliminary finding for our study was to assess the cell viability along with steroidogenesis upon B(a)P exposure. For that purpose dose and time dependent cell viability, testosterone production was checked. StAR being the major important molecule for testosterone production, cellular StAR protein expression at different dose and time point were also checked. Different doses of B(a)P (10, 20, 40, 50, and 100 μM) were studied for 48 hrs. Testosterone level in media was measured and western blots were performed in different groups. From the lower most dose, testosterone synthesis and StAR expression were significantly affected by B(a)P (Figure 2A). Time dependent effect of B(a)P on steroidogenesis in TM3 cells were studied with 20 μM dose for 0, 12, 24, 48, and 72 h. From 24 h the steroidogenesis was significantly (*p* ≤ 0.01–0.001) affected by B(a)P (Figure 2C).

MTT assay was performed to investigate the effect of B(a)P on TM3 Leydig cell viability. Results indicated that though cell viability was maintained above 50% at all concentrations of B(a)P, but the cell viability was gradually reduced with increasing concentrations of B(a)P (Figure 2B). Extended duration of treatment of B(a)P up to 72 h resulted significant cell death (*p* ≤ 0.01–0.001) numerically (Figure 2D).

Therefore, 20 μM B(a)P was chosen as the effective dose for the rest of the study as this particular dose did not induce any significant cell death on the other hand significantly altered the steroidogenic profile of Leydig cell.

### Resveratrol Prevents B(a)P Induced Steroidogenic Dysfunction *in vitro*

**Figure 3:**
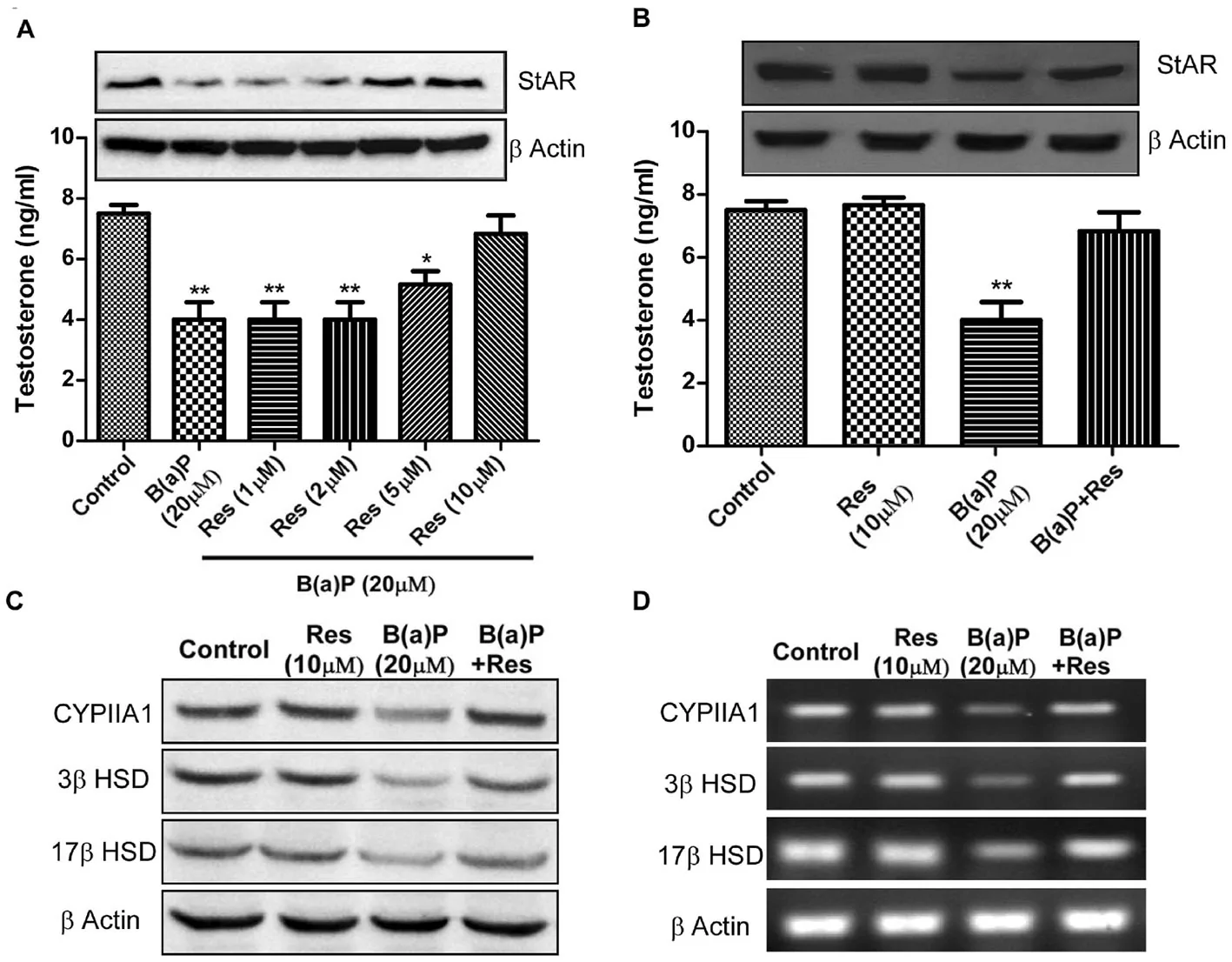
Dose dependent effect of Res on B(a)P induced steroidogenic dysfunction in TM3 cells. **(A)** Histogram expressing testosterone level in media and corresponding western blot of StAR (upper panel). **(B)** Histogram expressing testosterone level in media and corresponding western blot of StAR (upper panel) in TM3 cells. **(C)** Western blot and **(D)** RT-PCR of *CYP11A1, StAR, 3*β *HSD*, and *17*β *HSD* in TM3 cells. β Actin was used as the loading control. At least three independent experiments were performed. Histograms are expressing the value of, mean ± SEM. One-way ANOVA was followed by Dunnett multiple comparison test. The level of significance was set at ^**^(*P* ≤ 0.01–0.001); ^*^(*P* ≤ 0.01–0.05) as compared with control. B(a)P, Benzo(a)pyrene; Res, Resveratrol.

Till now our findings were based upon *in-vivo* studies. To delineate the mechanism of action of B(a)P in Leydig cell steroidogenic dysfunctions and possible protective action of Res, *in-vitro* study was the ultimate requisite. We have chosen normal mouse Leydig cell-line TM3 as *in-vitro* model. *In-vitro* study with TM3 cell were conducted to delineate the molecular mechanism of B(a)P induced steroidogenic dysfunction and preventive role of Res. Different doses of Res (1, 2, 5, 10 μM) were validated against 20 μM B(a)P for 48 hrs. Testosterone level in media was measured and western blot for StAR was performed in different groups (Figure 3A). These findings suggested 10 μM Res to be effective against 20 μM dose of B(a)P (Figures 3A,B).

Next we investigated the expression of steroidogenesis regulatory proteins like CYP11A1, 3β HSD and 17β HSD in TM3 cells upon B(a)P and Res treatment. Results suggested that Res significantly improved *in-vitro* steroidogenesis via modulating the protein and mRNA expression of *CYP11A1, 3*β *HSD*, and *17*β *HSD* (Figures 3C,D).

### Resveratrol Prevents B(a)P Induced ROS Generation and p38 MAPK Activation and Thus Maintains Leydig Cell Steroidogenesis

**Figure 4:**
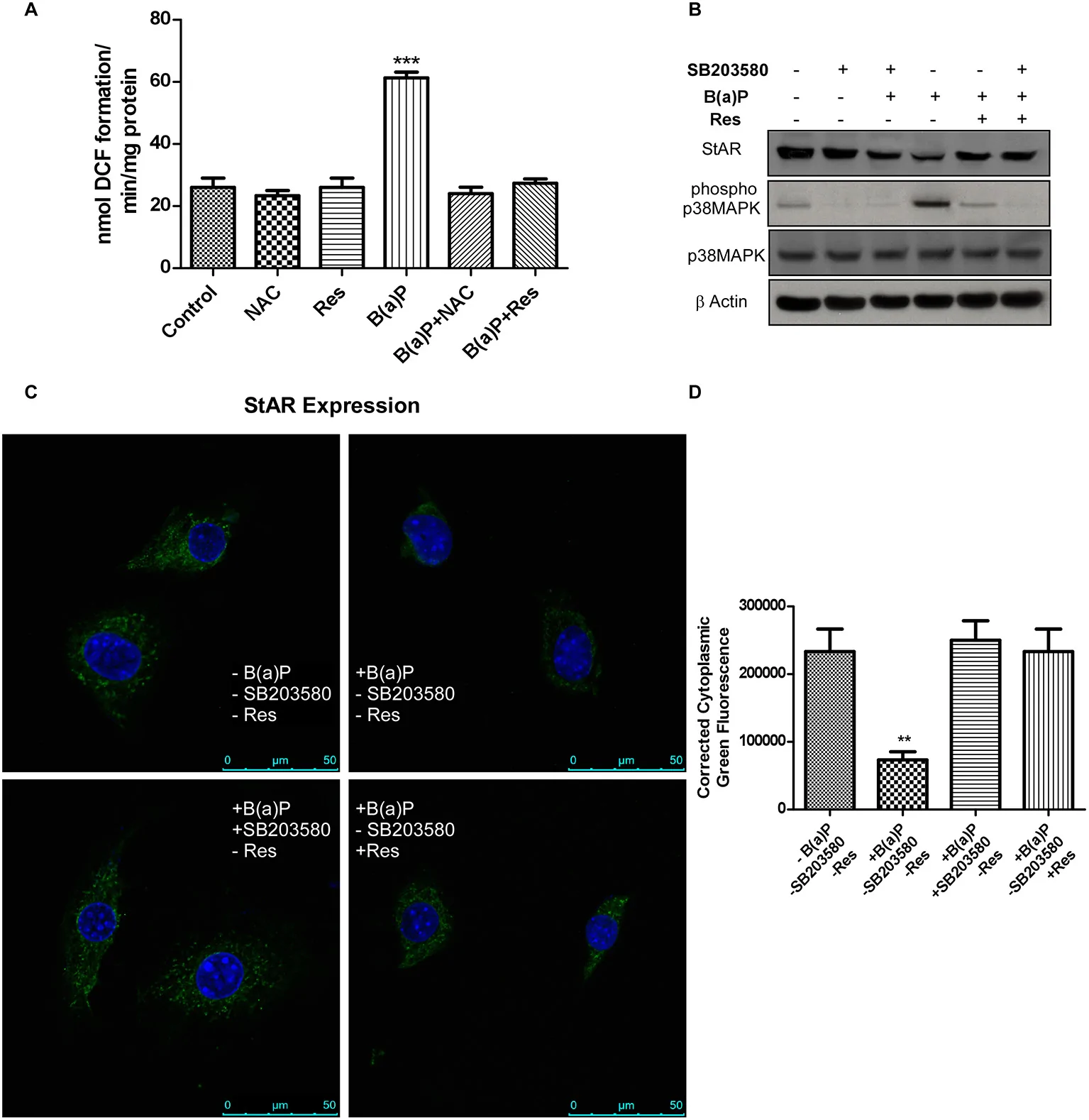
**(A)** Effect of B(a)P and Res on ROS generation in TM3 cells. Histogram is expressing the value of, mean ± SEM. One-way ANOVA was followed by Dunnett multiple comparison test. The level of significance was set at ^***^(*P* < 0.001); ^**^(*P* ≤ 0.01–0.001) as compared with control. **(B)** Western blot of StAR, phospho and complete p38 MAPK under different experimental conditions. β Actin was used as the loading control. **(C)** Immuno-cytochemistry of StAR expression in different experimental conditions. Nuclei were counter stained with DAPI (blue). Bar = 50 μm. Magnification = 600X. **(D)** Corrected fluorescence intensity of StAR protein was quantified and represented as bar diagram (Right panel). Pictures showed representative cells of each population and were representative of three independent experiments. The level of significance was set at ^***^(*P* < 0.001); ^**^(*P* ≤ 0.01–0.001) as compared with control. B(a)P, Benzo(a)pyrene; Res, Resveratrol; NAC,N Acetyl Cysteine.

The findings revealed that B(a)P exposure resulted ROS generation in TM3 cells. Further the potential ROS scavenging activity of Res in comparison to well-known anti-oxidant NAC (N Acetyl Cysteine) was checked. Experimental outcomes revealed that Res significantly prevented ROS generation in Leydig cells (Figure 4A). Oxidative stress induces p38 MAPK activation in different systems (21, 24). Oxidative stress induced activation of p38 MAPK has strong correlation with the Inhibition of steroidogenesis via the regulation of StAR (21, 24). We hypothesized that B(a)P induced p38 MAPK activation via ROS generation and activated p38 MAPK inhibited *StAR* gene expression. To strengthen our hypothesis cells were treated with p38 MAPK blocker SB203580 and ROS with p38 MAPK activation were checked. Western blot analysis revealed that blocking p38 MAPK significantly restored StAR expression (Figure 4B). This finding was reconfirmed by immune-cytochemistry (Figure 4C). Results showed that B(a)P induced decrease in cytoplasmic expression of StAR was retrieved with p38 MAPK blocker SB203580. And Res treatment decreased B(a)P induced activation of p38 MAPK along with restored StAR expression (Figures 4C,D). These findings provided the clear relation between the oxidative stress and steroidogenic dysfunction upon B(a)P exposure.

### Resveratrol Up-Regulates B(a)P Induced Alterations in the Gene Expression of Anti-oxidant Enzymes in Leydig Cells

**Figure 5:**
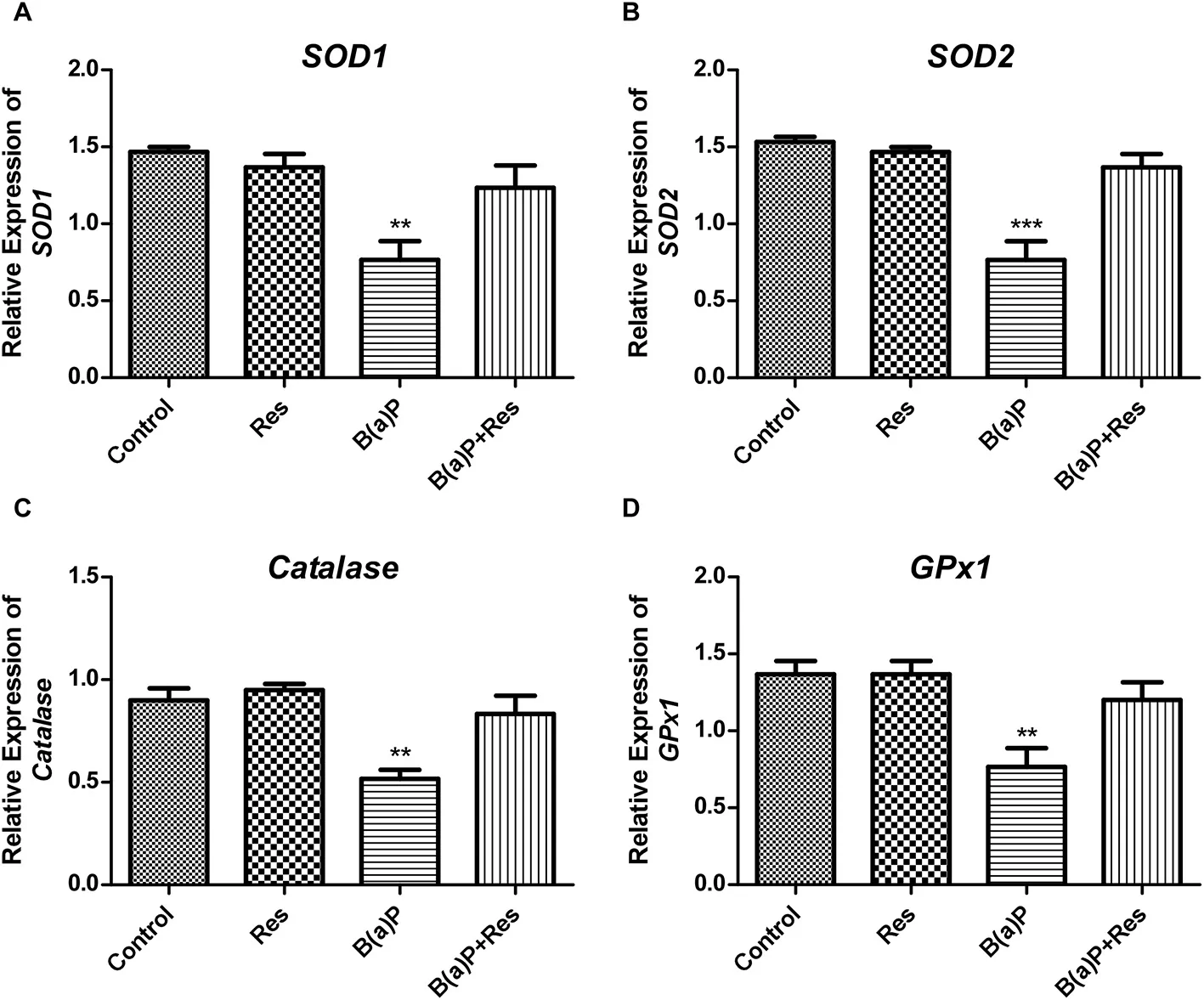
Effect of B(a)P and Res on the changes of the gene expression of anti-oxidant enzymes in isolated Leydig cells. qRT-PCR analyses of **(A)** *SOD1*, **(B)** *SOD2*, **(C)** *CAT*, and **(D)** *GPX1* mRNA levels. Histograms are expressing the value of, mean ± SEM. One-way ANOVA was followed by Dunnett multiple comparison test. The level of significance was set at ^***^(*P* < 0.001); ^**^(*P* ≤ 0.01–0.001) as compared with control. B(a)P, Benzo(a)pyrene; Res, Resveratrol.

ROS-induced oxidative stress is the disturbance in the regulation of balance between ROS production and the production of anti-oxidant enzymes. This anti-oxidant defense system is mainly comprised of antioxidant enzymes (e.g., *SOD1* [cytosolic CuZn-SOD (superoxide dis-mutase)], *SOD2* [mitochondrial Mn-SOD], *Catalase* [peroxisomal catalase] and *GPX1* [cytosolic/mitochondrial glutathione peroxidase 1]. ROS induces reduced functioning of these enzymes. Our previous findings revealed that B(a)P induced decrease in the activity of these enzymes in testis (5, 6). Herein we evaluated the mRNA expressions of *SOD1, SOD2, Catalase* and *GPX1*in isolated Leydig cells from B(a)P and Res treated animals. Our findings suggested that B(a)P treatment decreased the mRNA expression of *SOD1, SOD2, GPX*, and *Catalase* (Figures 5A–D). Whereas, Res treatment significantly up-regulated B(a)P induced alterations in the gene expression of anti-oxidant enzymes (Figure 5).

### Resveratrol Modulates Leydig Cell Steroidogenesis via Regulating StAR and SF1

**Figure 6:**
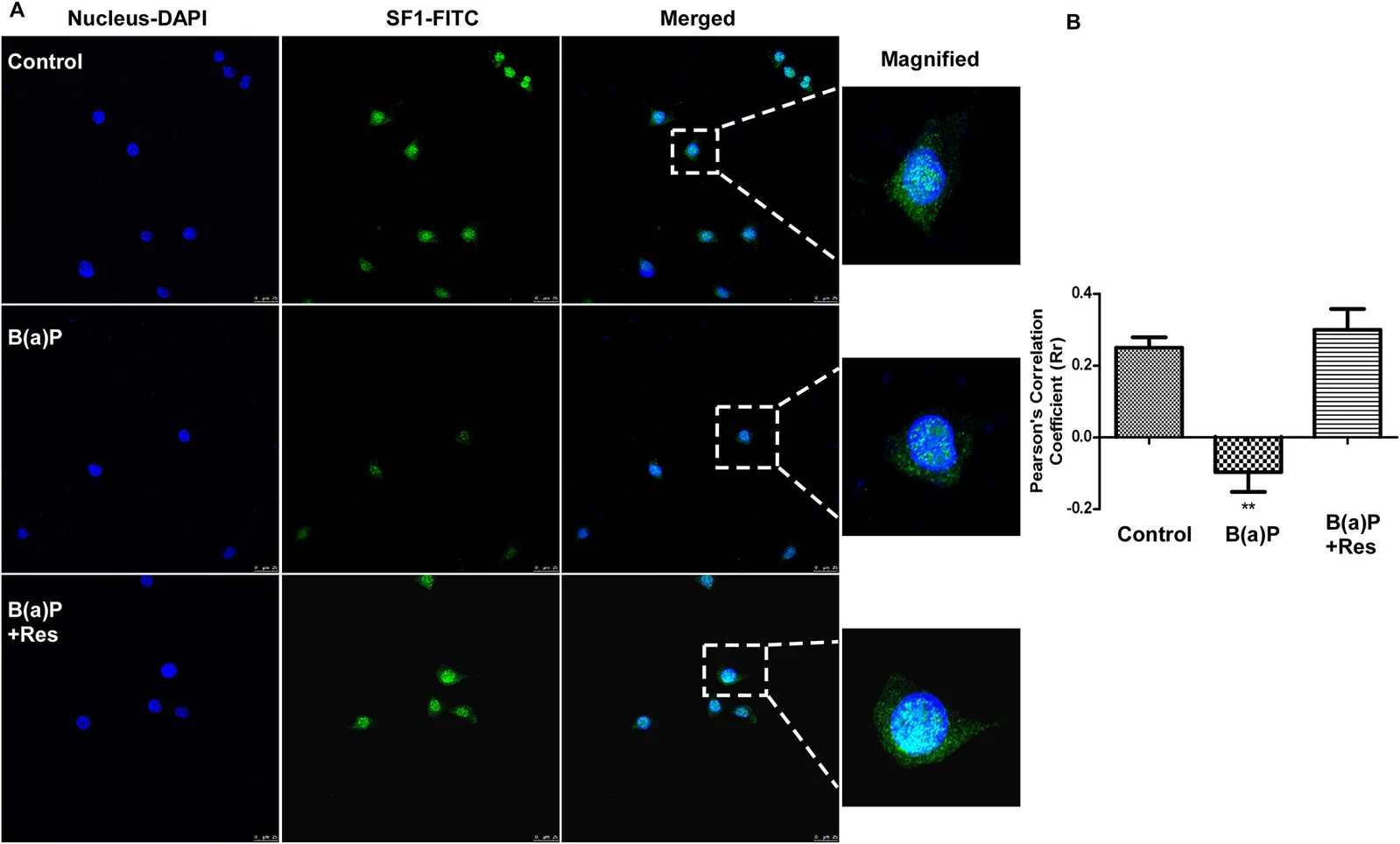
Immuno-cytochemical detection of SF1under the influence of B(a)P and Res in primary Leydig cells. **(A)** Representative fluorescence images showed the association between SF1 (FITC/green) and nuclei (DAPI/blue). Enlargement of the areas indicated localization of SF1 in nucleus. Pictures showed representative cells of each population and were representative of three independent experiments. Bar = 25 μm. Magnification 400x. **(B)** The Pearson's coefficients determining the level of overlap of FITC and DAPI under different experimental groups has been represented graphically. The level of significance was set at ^**^(*P* ≤ 0.01–0.001) as compared with control. B(a)P, Benzo(a)pyrene; Res, Resveratrol.

StAR is a major steroidogenesis regulatory protein in Leydig cells. There are several factors involved with the activity of StAR promoter. Steroidogenic Factor 1 (SF-1/Ad4BP) is a transcription factor whose activity is exerted by binding to the promoter of the *StAR* gene (25). SF-1 is highly expressed in steroidogenic cell types where it functions to help control the tissue-specific expression of genes involved in the steroid hormone biosynthesis pathway (26). StAR promoter activity is regulated by the combinative function of different transcription factors. We have found that B(a)P induced decrease in SF1 expression in Leydig cells. Immuno-cytochemistry in isolated Leydig cells revealed that, being a transcription factor its expression was majorly localized in nucleus. And the nuclear expression of SF1 was improved with Res treatment (Figure 6).

**Figure 7:**
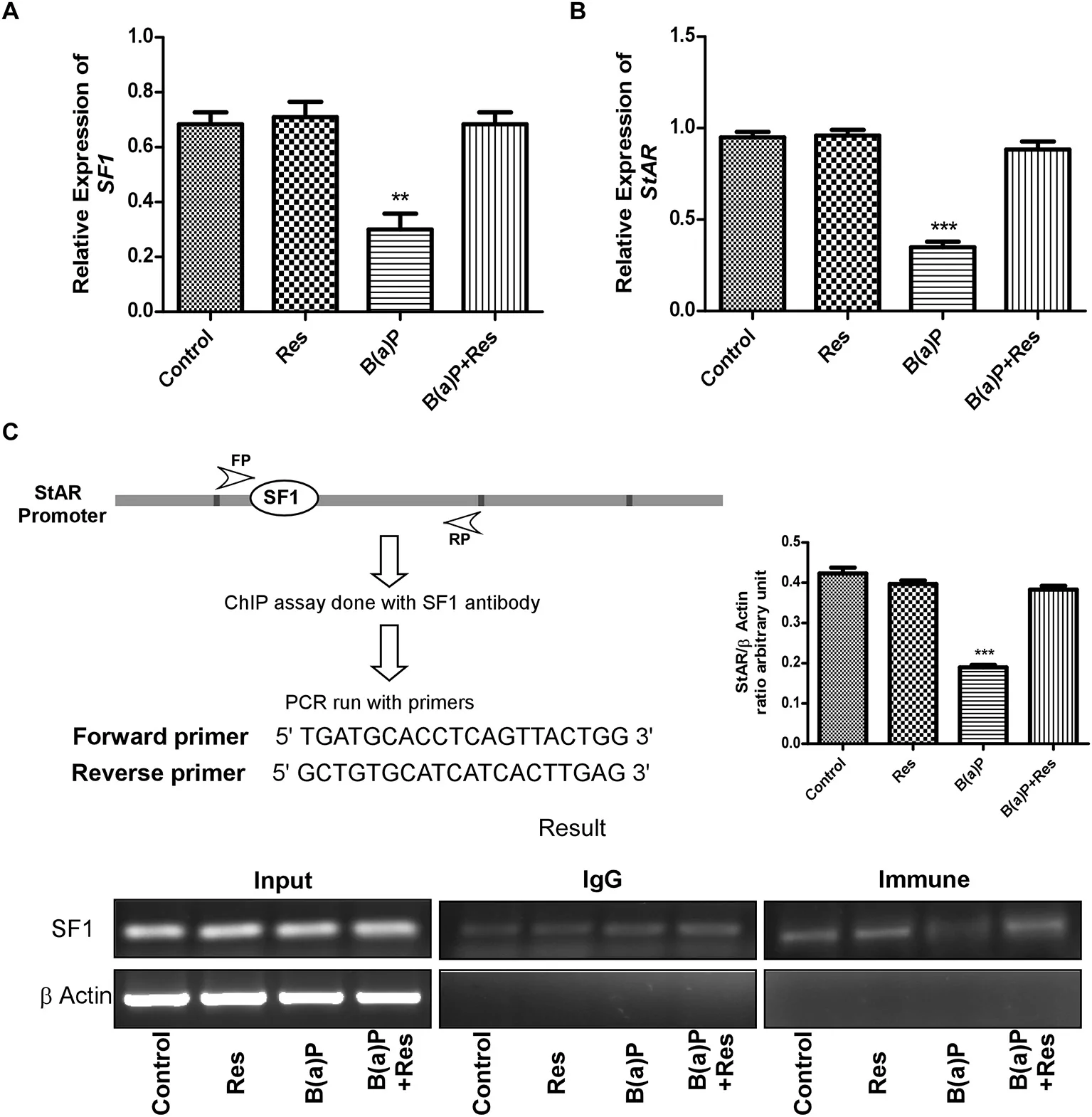
Quantitative real time PCR analysis of **(A)** *SF1* and **(B)** *StAR* expression. **(C)** Schematic representation of the *StAR* promoter and the sequence of the forward and reverse primer used for ChIP analysis. ChIP assay in isolated Leydig cells treated with B(a)P and Res determining the recruitment of SF1 to the StAR promoter. The corresponding histogram exhibit the densitometric quantification of ChIP result, expressing mean ± SEM of relative arbitrary units. One-way ANOVA was followed by Dunnett multiple comparison test. The level of significance was set at ^***^(*P* < 0.001); ^**^(*P* ≤ 0.01–0.001) as compared with control. B(a)P, Benzo(a)pyrene; Res, Resveratrol.

Further findings revealed that B(a)P exposure decreased the mRNA expression of StAR and SF1 in isolated Leydig cells and Res significantly increased the mRNA expression of StAR and SF1 (Figures 7A,B). ChIP assay with the proximal promoter region of StAR with SF1 antibody in Leydig cells exhibited that B(a)P treatment decreased the SF1 recruitment to StAR promoter; whereas Res treatment enhanced the SF1 recruitment (Figure 7C). These findings propose the role of SF1 as an active target molecule in B(a)P induced steroidogenic dysfunction.

**Figure 8:**
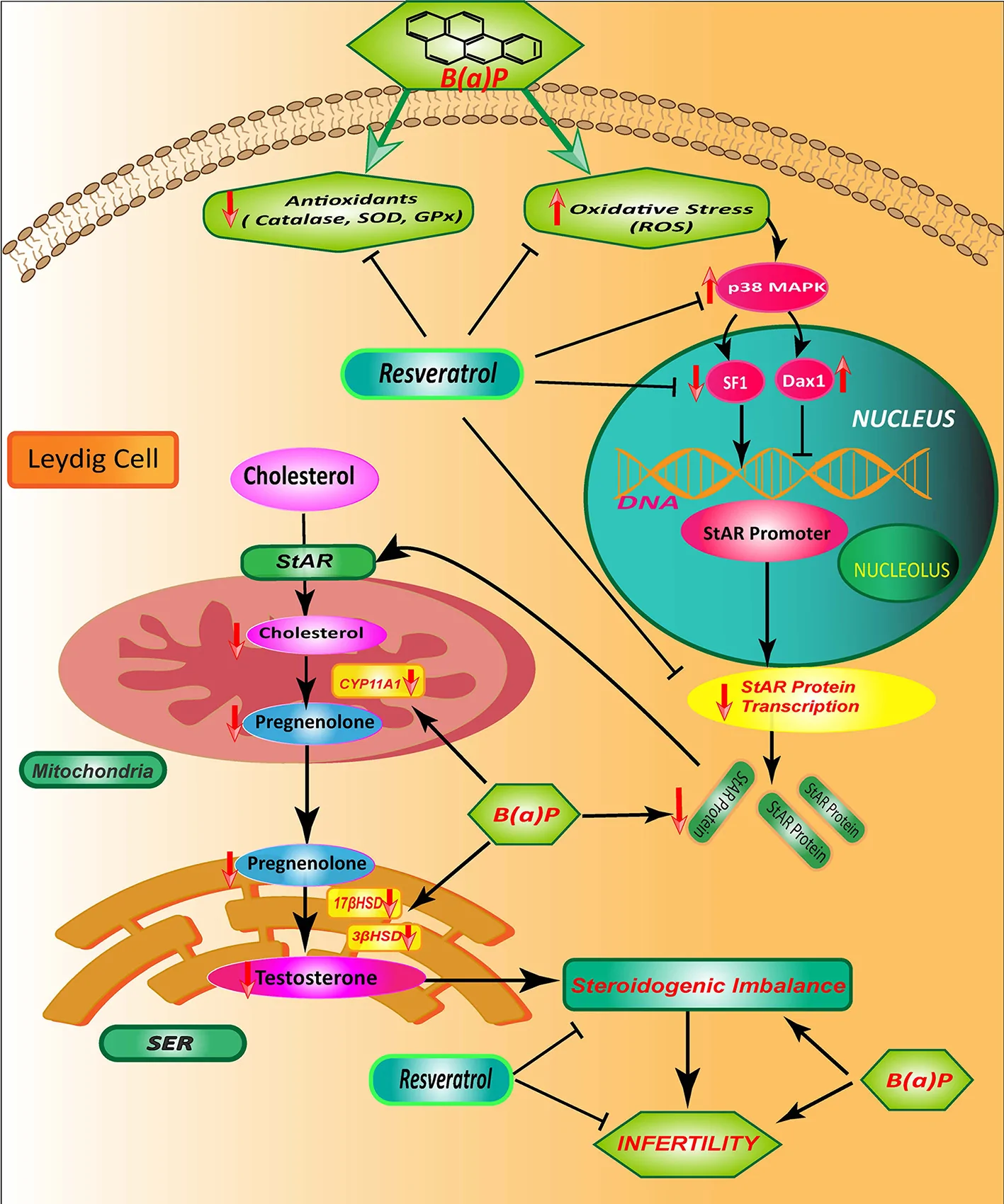
Schematic diagram showing the mode of protective action of Res on B(a)P induced Leydig cell steroidogenic dysfunction. B(a)P generates oxidative stress, activates p38 MAPK and decreases StAR protein production; that cumulatively impairs the testicular steroidogenesis. Res prevent oxidative stress and associated p38 MAPK activation. Res administration also improves StAR expression and SF1 binding to StAR promoter. Thus, Res protects against B(a)P induced down-Regulation of Leydig cell steroidogenesis that involves MAPK activation and modulation of SF1. B(a)P, Benzo(a)pyrene; Res, Resveratrol.

These results altogether concluded that B(a)P induced steroidogenic suppression is a combinatorial effect of oxidative stress, p38MAPK activation, transcriptional down-regulation of StAR via SF1 (Figure 8).

## Discussion

Benzo(a)pyrene [B(a)P] is a common environmental toxicant that is generated from several natural (forest fire, volcanic explosion etc.) and anthropological sources (tobacco smoke, vehicular exhaust, industrial smoke etc.). With the advancement of human civilization, exposure of environmental PAH like B(a)P has increased significantly and general population are exposing low to moderate level of B(a)P. This leads the male reproductive health under constant threat.

Our previous findings suggested that B(a)P induces apoptosis in testicular spermatogonial germ cell population. But surprisingly B(a)P cannot induce apoptosis in the steroidogenic Leydig cells but, testicular steroidogenesis is hindered. These findings persuade us to unravel the molecular crosstalk behind B(a)P induced disturbance in steroidogenic mechanism. Our previous findings proposed resveratrol (Res) as a potential warrior against B(a)P. Herein the mode of action of Res against B(a)P in steroidogenic dysfunction in Leydig cell was investigated.

Leydig cells comprise the steroidogenic compartment of testis. In adult testis these cells possess specialized properties to proliferate rarely and being less susceptible to apoptosis (27).

Our previous findings suggested that B(a)P exposure induce apoptosis particularly to the testicular germ cell population (5, 6). This finding is consistent with previous report (11). Our findings reveal that Leydig cells express very poor level of CYP1A1. CYP1A1 is the major molecule that is involved with the metabolic modulation of B(a)P to its toxic form (28). Therefore, we presume that in testis B(a)P execute exclusive molecular signaling in Leydig cells.

Leydig cells are very much sensitive to oxidative stress and they possess strong anti-oxidant mechanism to fight with the oxidative stress (29). Our findings documented that B(a)P exposure induced ROS generation in Leydig cells as well as transcriptional regulation of antioxidant defensive enzymes were deregulated. There is a strong correlation between the oxidative stress and testicular steroidogenesis (29). Herein B(a)P induced decease in circulating and intra-testicular testosterone level strengthened the concept. Along with that the translational and transcriptional level of steroidogenesis regulatory molecules (CYP11A1, 3β HSD, 17β HSD), and StAR were significantly altered in Leydig cells. B(a)P induced steroidogenic dysfunction particularly altering anti-oxidative defense system in testicular Leydig cells. Our findings are in accordance with previous observations that ROS mediated excessive oxidative stress inhibits steroidogenesis (24, 29).

p38 MAPK is the stress induced kinase which is activated by oxidative stress and has been shown to be activated by ROS in testis and its activation induce damage in anti-oxidant mechanism of cell that in turn increase ROS generation (30–32). Activation of the p38 MAPK signaling pathway is functionally linked to the oxidative stress response and mediates its inhibitory effect on steroidogenesis (24, 33). Our findings indicated that B(a)P exposure induces oxidative stress and p38 MAPK activation in Leydig cells. And this p38 MAPK activation is triggered by the oxidative stress and decrease in the transcriptional regulation of anti-oxidant enzymes (SOD1, SOD2, Catalase and GPx1). The findings also revealed that B(a)P exposure induced activation of p38 MAPK and inhibited steroidogenesis in Leydig cells. StAR being the major target for B(a)P induced steroidogenic dysfunction (4, 6), we tried to find out the link between StAR and p38MAPK activation under the influence of B(a)P induced oxidative stress. *In vitro* studies showed that blocking the activity of p38 MAPK significantly restored StAR expression, in the presence of B(a)P. This established that B(a)P induced StAR mediated steroidogenic dysfunction is linked with the activation of p38 MAPK.

Our selection for Resveratrol (Res) was based upon its natural ability to act as an anti-oxidant and AhR antagonist has been supported by ours as well as others in previous studies (6, 22, 34). Herein our results established that Res being an anti-oxidant effectively prevented oxidative stress in Leydig cells. Beside that Res prevented B(a)P induced p38 MAPK activation and restored StAR expression and/or steroidogenesis.

StAR protein plays the pivotal role in the acute phase of steroidogenesis, mediating the rate-limiting translocation of cholesterol from the outer to the inner mitochondrial membrane, where side-chain cleavage enzyme (P450scc; Cyp11A) carries out the first committed step in steroidogenesis (35), i.e., conversion of cholesterol to pregnenolone. StAR is the major target of most of the endocrine disrupting compounds (10, 36). The transcriptional regulation of *StAR* gene is complex with numerous factors involved in StAR transcription. Among several transcription factors that regulate StAR expression, SF1 and DAX1 are the most common players (14). SF1 is an important factor for the proper functioning of StAR promoter. Analysis of the StAR promoter region in the rat, mouse, and human has revealed the presence of multiple SF-1 binding sites (18). B(a)P treatment prevented the recruitment of SF1 to the StAR proximal promoter region. Whereas, Res treatment was increased the SF1 recruitment to the promoter. At the same time western blot and immune-cytochemical findings exhibited that B(a)P down-regulate the SF1 expression and Res co-treatment significantly improved SF1 expression. Whereas, DAX1 also represses the promoter activity of several steroidogenic genes including *StAR, Cyp11A, Cyp19* etc. (35). In StAR promoter DAX1 exerts its function via its antagonistic activity with SF1.

Experimental outcomes thus suggested that B(a)P exposure induced oxidative stress and steroidogenic dysfunction in Leydig cells. Res effectively prevented ROS generation and p38 MAPK activation and thus protected steroidogenesis in Leydig cells and conserved male reproductive health.

Altogether, the data indicate that B(a)P interrupted Leydig cell steroidogenesis via down-regulating StAR expression. B(a)P exposure resulted oxidative stress mediated activation of stress kinase p38 MAPK. Experimental findings showed the inhibitory effect of p38 MAPK on Leydig cell steroidogenesis in *in-vivo* and *in-vitro* through the decrease in StAR gene expression. Furthermore, B(a)P induced decrease in SF1 expression could also play an important role in this molecular interplay. Res being a potent anti-oxidant ameliorated the B(a)P induced p38 MAPK mediated inhibition of StAR gene transcription and diminution of steroidogenesis.

Our findings suggested that B(a)P treatment activated oxidative stress induced p38MAPK in both primary and TM3 Leydig cells. And subsequently impaired steroidogenesis by the modulation of StAR expression (Figure 8). SF1 is another major player which is associated with the regulation of the promoter activity of StAR. In recent times many natural dietary phytochemicals have been selected for clinical studies. These compounds typically involve multiple signaling transduction pathways. They themselves or their synthetic analogs have several aspects of research. From our findings it can be concluded that Resveratrol may turn up with new hope in the field of environmental toxicity induced reproductive dysfunctions. However, there are several developmental challenges need to be overcome to establish it as a functional molecule. Moreover, there is not enough evidence to recommend the consumption of resveratrol to combat against B(a)P induced toxicity in human. Further human clinical trials are needed to establish the exact dosage requirements of resveratrol against environmental reproductive toxicants.

## Ethics Statement

Institutional Animal Ethics Committee of Bose Institute, Approval No. IAEC/BI/30/2015.

## Author Contributions

BB, SC, PC, and KJ conceived, designed, and performed the experiments. BB, PC, DG, and KJ wrote the manuscript.

### Conflict of Interest Statement

The authors declare that the research was conducted in the absence of any commercial or financial relationships that could be construed as a potential conflict of interest.
