# Correction: Protective effect of resveratrol on benzo(a)pyrene induced dysfunctions of steroidogenesis and steroidogenic acute regulatory gene expression in Leydig cells

**DOI:** 10.3389/fendo.2026.1946101

**Published:** 2026-09-09

**Authors:** Bhaswati Banerjee, Supriya Chakraborty, Pratip Chakraborty, Debidas Ghosh, Kuladip Jana

**Affiliations:** 1 Division of Molecular Medicine, Bose Institute, Calcutta Improvement Trust Scheme VIIM, Kolkata, India; 2 Department of Infertility, Institute of Reproductive Medicine, Kolkata, India; 3 Department of Bio-Medical Laboratory Science and Management, Vidyasagar University Midnapore, Midnapore, India

**Keywords:** B(a)P, leydig cell, ROS, StAR, p38 MAPK, SF1

After publication of this article, concerns were raised on PubPeer regarding **Figures 2A**, **3A**: https://pubpeer.com/publications/6A243DAD043D3E0AAE1831B0A4C136

**Figure 2:**
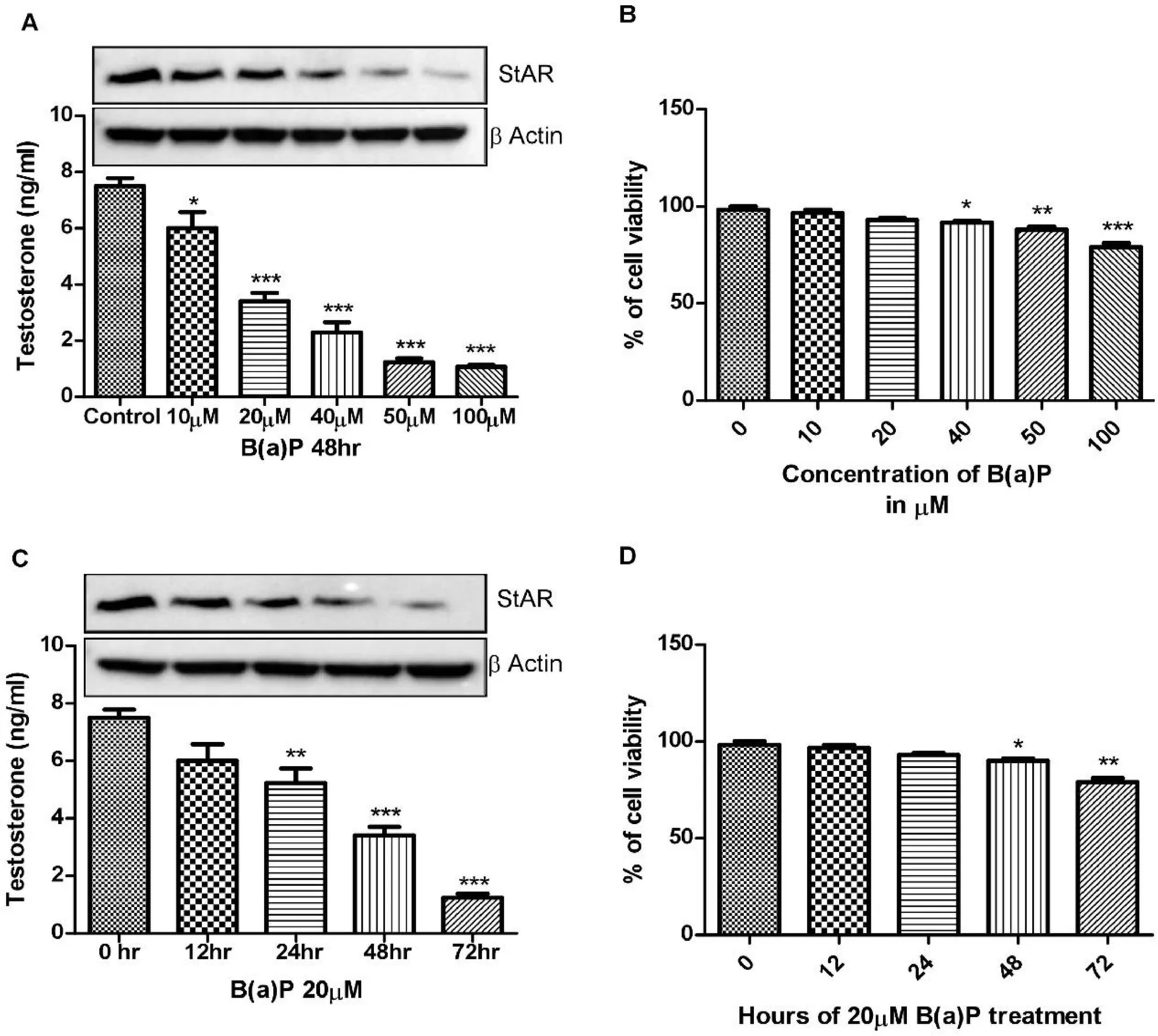
**(A)** Dose dependent effect of B(a)P (10–100 μM) on steroidogenesis in TM3 cells for 48 h. Histogram expressing testosterone level (ng/ml) in media and corresponding Western blot of StAR (upper panel). **(B)** Histogram exhibiting cell viability after treatment with increasing concentrations of B(a)P (10–100 μM) in TM3 cells for 48 h. **(C)** Time dependent effect of B(a)P on steroidogenesis in TM3 cells. Histogram expressing testosterone level (ng/ml) in media and corresponding Western blot of StAR (upper panel). **(D)** Histogram representing cell viability after treatment with increasing concentrations of B(a)P at different time points with 20 μM B(a)P in TM3 cells. β Actin was used as the loading control. Histograms are expressing the value of mean ± SEM. One-way ANOVA was followed by Dunnett multiple comparison test. The level of significance was set at ***(P < 0.001); **(P ≤ 0.01–0.001); *(P ≤0.01–0.05) as compared with control. B(a)P, Benzo(a)pyrene.

**Figure 3:**
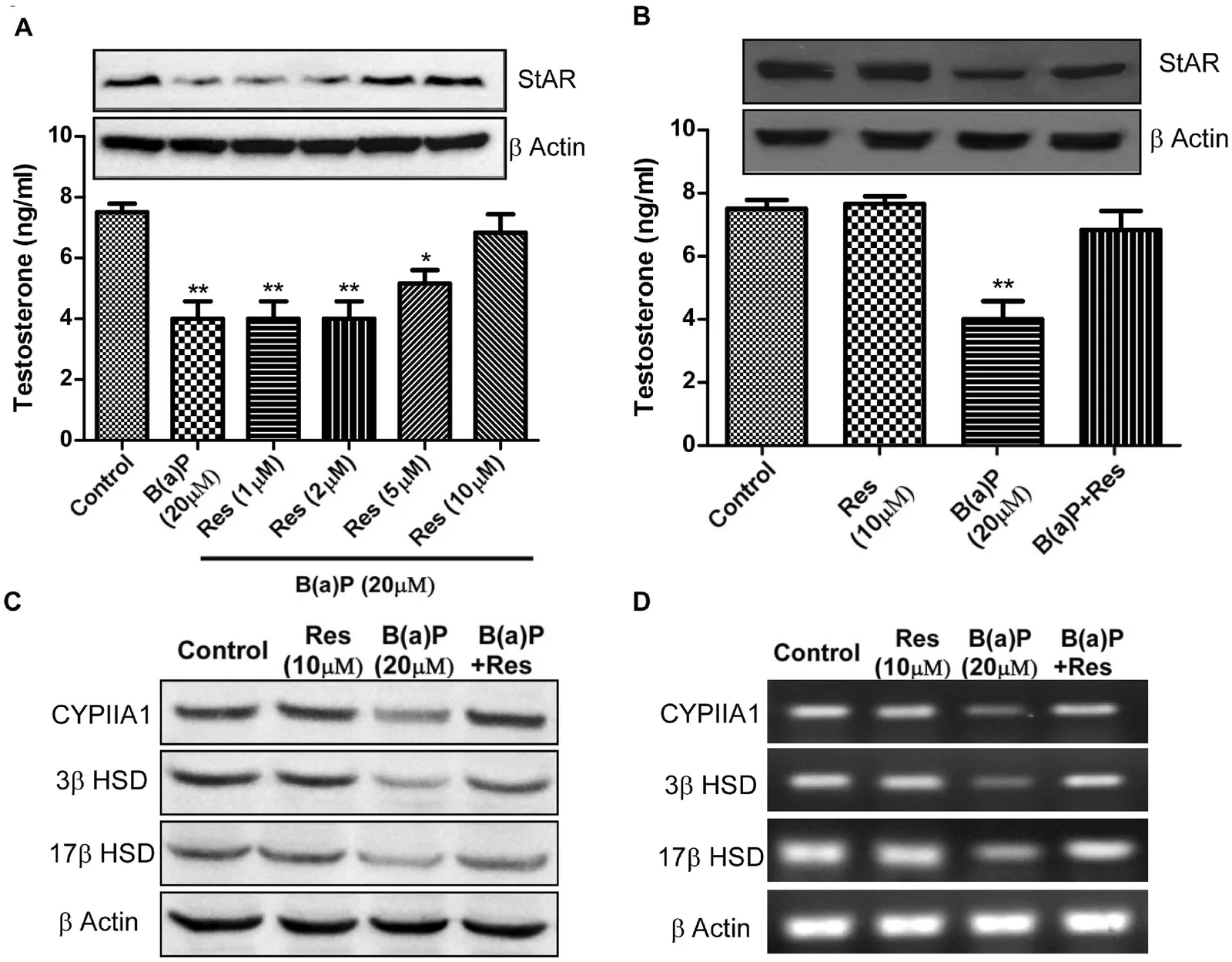
Dose dependent effect of Res on B(a)P induced steroidogenic dysfunction in TM3 cells. **(A)** Histogram expressing testosterone level in media and corresponding Western blot of StAR (upper panel). **(B)** Histogram expressing testosterone level in media and corresponding Western blot of StAR (upper panel) in TM3 cells. **(C)** Western blot and **(D)** RT-PCR of *CYP11A1, StAR, 3*β *HSD*, and *17*β *HSD* in TM3 cells. β Actin was used as the loading control. Histograms are expressing the value of, mean ± SEM. One-way ANOVA was followed by Dunnett multiple comparison test. The level of significance was set at **(*P* ≤ 0.01–0.001); *(*P* ≤ 0.01–0.05) as compared with control. B(a)P, Benzo(a)pyrene; Res, Resveratrol.

As the experiments described in the article were performed in 2015-2016, the original uncropped, full-scan membrane files of 2016 are no longer retrievable. The authors repeated all the pertinent Western Blot experiments underlying **Figures 2a, c**, and **3a** in their entirety. The corrected **Figures 2** and **3** appear below.

The original article has been updated.

